# The effects of preventative cannabidiol in a male *neuregulin 1* mouse model of schizophrenia

**DOI:** 10.3389/fncel.2022.1010478

**Published:** 2022-11-03

**Authors:** Gabriela Visini, Samara Brown, Katrina Weston-Green, Cynthia Shannon Weickert, Rose Chesworth, Tim Karl

**Affiliations:** ^1^School of Medicine, Western Sydney University, Campbelltown, NSW, Australia; ^2^School of Medical, Indigenous and Health Sciences, Faculty of Science, Medicine and Health, University of Wollongong, Wollongong, NSW, Australia; ^3^Illawarra Health and Medical Research Institute, University of Wollongong, Wollongong, NSW, Australia; ^4^Neuroscience Research Australia, Sydney, NSW, Australia

**Keywords:** cannabidiol (CBD), Δ9-tetrahydrocannabinol (THC), behavior, brain pathology, *neuregulin 1*

## Abstract

Cannabidiol (CBD) is a non-intoxicating cannabinoid with antipsychotic-like properties, however it’s potential to prevent schizophrenia development has not been thoroughly investigated. Brain maturation during adolescence creates a window where CBD could potentially limit the development of schizophrenia. The *neuregulin 1 transmembrane domain* heterozygous (*Nrg1 TM* HET) mutant mouse shows face, predictive, and construct validity for schizophrenia. Here we sought to determine if CBD given in adolescence could prevent the development of the schizophrenia-relevant phenotype, as well as susceptibility to the psychoactive cannabinoid Δ^9^-tetrahydrocannabinol (THC) in *Nrg1 TM* HET mice. Adolescent male *Nrg1* mutants and wild type-like (WT) animals were administered 30 mg/kg CBD i.p. daily for seven weeks, and were tested for locomotion, social behavior, sensorimotor gating and cognition, and sensitivity to acute THC-induced behaviors. GAD67, GluA1, and NMDAR1 protein levels were measured in the hippocampus, striatum, and prefrontal cortex. Chronic adolescent CBD increased locomotion in animals regardless of genotype, was anxiolytic, and increased social behavior when animals were tested for their acute THC response. CBD did not alleviate the schizophrenia-relevant hyperlocomotive phenotype of *Nrg1* mutants, nor deficits in social behaviors. *Nrg1* mutant mice treated with CBD and THC showed no habituation to a startle pulse, suggesting CBD increased vulnerability to the startle habituation-reducing effects of THC in mutant mice. CBD increased levels of GluA1, but reduced levels of GAD67 in the hippocampus of *Nrg1* mutants. These results suggest adolescent CBD is not effective as a preventative of schizophrenia-relevant behavioral deficits in mutants and may actually contribute to pathological changes in the brain that increase sensitivity to THC in particular behavioral domains.

## Introduction

Current treatments for schizophrenia do not have a high adherence rate (Higashi et al., 2013; Sendt et al., 2015) and can lead to several adverse health conditions (e.g., heart disease and metabolic syndrome) (Lambert et al., 2004; Tschoner et al., 2007), demonstrating a need for novel treatments and/or treatment approaches. A novel focus of schizophrenia research is identifying factors that may help to prevent disease onset especially during adolescence (Heresco-Levy, 2011). Adolescence is an important period of neurodevelopment which is sensitive to external factors, such as exposure to drugs of abuse and psychosocial stress (Winters and Arria, 2011; Gomes and Grace, 2017). This maturational period is also a potential window for pharmacological intervention aimed at preventing the development of mental disorders such as schizophrenia (Heresco-Levy, 2011). In preclinical studies, early life interventions with environmental enrichment (such as altered housing conditions) can ameliorate schizophrenia-relevant behavioral domains in animals, and in clinical studies, neuroprotective agents when administered in adolescence can improve symptoms in schizophrenia patients (Bossong and Niesink, 2010; do Prado et al., 2016; Takahashi and Suzuki, 2018).

The phytocannabinoid cannabidiol (CBD) is currently investigated as a remedial treatment option for schizophrenia (Schoevers et al., 2020) but it is possible that CBD may also have preventative properties (Chesney et al., 2021) in line with what has been demonstrated in other neurological animal models (Hayakawa et al., 2008; Cheng et al., 2014; Loss et al., 2020). Adolescence may be an appropriate period for preventative CBD treatment in schizophrenia, as some preclinical research suggests that CBD treatment during mid-adolescence can attenuate schizophrenia-relevant behaviors (Osborne et al., 2017, 2019a). Furthermore, short-term 7-day CBD treatment of clinical populations at risk of developing psychosis can attenuate social stress (Wilson et al., 2019; Appiah-Kusi et al., 2020), suggesting CBD may have the ability to alter the trajectory of psychosis development. However, no studies have investigated the effects of long-term adolescent CBD treatment in at-risk populations or mice that carry genetic predisposition for schizophrenia.

Cannabis misuse during adolescence is a component risk factor for schizophrenia development (Henquet et al., 2008; Fernandez-Espejo et al., 2009; Bossong and Niesink, 2010). Interestingly, some studies show that cannabis with higher levels of CBD than THC induces fewer psychosis-related experiences in non-clinical populations than cannabis with higher levels of THC than CBD (Schubart et al., 2011; Solowij et al., 2019). In addition, acute CBD can reduce THC-induced social withdrawal and cognitive impairment in rats (Malone et al., 2009). Thus, it is possible that preventative long-term CBD treatment could affect the neuro-behavioral effects of THC, and we will address this question in the present study.

*Neuregulin 1* (*NRG1*) is a well-established genetic risk factor for schizophrenia (Mostaid et al., 2016), and a mutation in the transmembrane domain region of *NRG1* is found in patients with schizophrenia (Walss-Bass et al., 2006). A multitude of previous studies, including from our team, have established face, construct, and predictive validity for *Nrg1 transmembrane domain* heterozygous (*Nrg1 TM* HET) mice (review: Karl, 2013), which exhibit age-dependent and sex-specific differences in locomotion, sensorimotor gating and social behaviors (Karl et al., 2007; van den Buuse et al., 2009; Desbonnet et al., 2017) changes to glutamatergic and GABAergic signaling and inflammatory tone (Desbonnet et al., 2012; Long et al., 2012; Newell et al., 2013; Chohan et al., 2014) as well as altered sensitivity to THC (Boucher et al., 2007; Long et al., 2013), reflecting findings in clinical cohorts (Saha et al., 2005; Han et al., 2012). Specifically, *Nrg1 TM* HET males exhibit hyperlocomotion in the open field (Karl et al., 2007), deficits in social interaction (SI) (O’Tuathaigh et al., 2008), and deficits in prepulse inhibition (PPI) (Stefansson et al., 2002). Furthermore, *Nrg1* mutants display altered sensitivity to cannabinoids in adolescence (Long et al., 2013) which may suggest a window for utilizing the protective effects of particular cannabinoids. Female mutants do not exhibit a phenotype of the same strength (Long et al., 2010a; Chesworth et al., 2012), which corresponds with reduced symptom severity and improved responses to antipsychotic medication observed in women with schizophrenia (Leung and Chue, 2000).

Here, we investigated if adolescent CBD treatment could prevent the development of schizophrenia-relevant behaviors in *Nrg1 TM* HET males, and also reduce susceptibility to an acute THC challenge in early adulthood. We assessed GAD67, GluA1 and NR1 protein levels in the hippocampus, striatum and prefrontal cortex (PFC) as an index of glutamatergic and GABAergic function in schizophrenia-relevant brain regions to determine neurochemical consequences of chronic adolescent CBD treatment.

## Materials and methods

### Animals

Male *Nrg1 TM* HET and non-mutant wild type-like (WT) male littermates were bred and group housed in individually ventilated cages (Type Mouse Version 1: Airlaw, Smithfield, Australia) at Animal BioResources (Moss Vale, NSW, Australia). Female *Nrg1 TM* HET animals do not show an as pronounced schizophrenia-relevant phenotype as male animals (Long et al., 2010a), which reflects some studies showing more severe symptoms in male patients versus female patients (Leung and Chue, 2000; Saha et al., 2005). Likewise, the differential cannabinoid sensitivity is more pronounced in male *Nrg1* mutant animals (Boucher et al., 2007; Long et al., 2010a,^2013^). For this reason, only male animals were chosen for this study.

At approximately 21–30 days old mice were transported to the mouse holding and test facilities at the School of Medicine, Western Sydney University (WSU), and were transferred to group-housing in filter top cages (1144B: Techniplast, Rydalmere Australia) with corn cob bedding (Tecniplast Australia, Rydalmere, Australia) and tissues for nesting material. Mice were kept in a 12:12 h light:dark schedule [light phase: white light (illumination: 124 lx), dark phase: red light (illumination: < 2 lx; light phase from 0900 to 2100). Mice were fed *ad libitum* with mouse feed pellets (Gordon’s Specialty Stockfeeds Pty Ltd., Yanderra, NSW, Australia) and water. Age-matched adolescent male A/J mice from Animal Resources Centre (Canning Vale, WA, Australia) were used as conspecifics in the social interaction test. All research projects were approved by the WSU Animal Care and Ethics Committee (#A11746, A13298) and were in accordance with the Australian Code of Practice for the Care and Use of Animals for Scientific Purposes.

### Drug preparation and administration

Powdered cannabidiol (CBD: THC Pharm GmbH, Frankfurt/Main, Germany) was prepared as published previously (Long et al., 2012), being dissolved in equal parts of Tween 80 (Sigma-Aldrich Co, St Louis, MO, USA) and 100% ethanol. It was then diluted with 0.9% sodium chloride to the final concentration (5% ethanol, 5% Tween 80, 90% saline, final conc. of 0.3% CBD/3 mg/ml). A vehicle control (VEH) was prepared by mixing all components minus CBD. VEH and CBD at 30 mg/kg bodyweight were injected daily intraperitoneally (i.p.); the injection volume was 10 ml (Long et al., 2012). Treatment started on PND35 (±5 days), where mice were injected once daily in the afternoon (1200–1500) for three weeks (see Table 1 for experimental timeline), after which behavioral testing commenced. Injections continued while behavioral testing was performed. During the baseline behavioral testing period, animals were injected after the relevant tests concluded for the day in order to avoid possible effects of acute CBD administration. CBD or VEH treatment ended approximately 24 h before animals were euthanized. Mice were weighed every four days and dosage of CBD was adjusted accordingly.

**TABLE 1:** Testing timeline and animal numbers: Test order, age (days), and animal numbers per group (*n)*.

Test order				Postnatal age (days)			
Adolescent CBD Treatment				35–84 (±5 days)			
Open field (OF)				56 (±5 days)			
Social interaction (SI)				58–59 (±5 days)			
Prepulse inhibition (PPI)				60–64 (±5 days)			
Fear conditioning (FC)				65–68 (±5 days)			
THC challenge (OF, SI, PPI)				77 (±5 days)			
Tissue collection				85 (±5 days)			

**Animal numbers**							

**WT**				***Nrg1 TM* HET**			
**VEH (14)**		**CBD (15)**		**VEH (23)**		**CBD (19)**	
**VEH**	**THC**	**VEH**	**THC**	**VEH**	**THC**	**VEH**	**THC**

7	7	7	8	11	12	9	10

Treatments included 30 mg/kg cannabidiol (CBD), 3 mg/kg Δ^9^-tetrahydrocannabinol (THC), or vehicle (VEH).

For the THC challenge, THC and VEH (THC Pharm GmbH, Frankfurt/Main, Germany) were prepared similarly to CBD preparation and in accordance with previous THC preparations in our laboratory (Boucher et al., 2007; Long et al., 2013). An acute dose of 3 mg/kg of THC was injected intraperitoneally to both VEH and CBD groups 30 minutes before behavioral testing commenced, at approximately 77 days (±5 days) of age. As with previous days, CBD was given at the end of the testing day. The THC dose was based on previous research in our laboratory (Boucher et al., 2007) as well as pilot experiments.

### Behavioral testing

**FIGURE 1:**
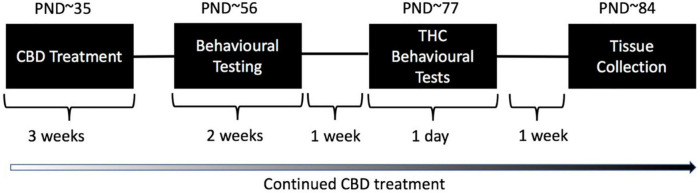
Treatment and testing schedule for test animals: Wild type-like (WT) and *neuregulin 1 transmembrane domain* heterozygous (*Nrg1 TM* HET) mice were treated chronically with either vehicle (VEH) or 30 mg/kg cannabidiol (CBD). Daily treatment commenced on post-natal day (PND) 35 ± 5. Mice commenced behavioral evaluation (starting PND 56 ± 5 days) while CBD treatment continued post-testing. Effects of chronic adolescent CBD treatment on behavior were evaluated between PND 56–70 ± 5. An acute THC challenge (3 mg/kg or VEH prior to testing) was administered on PND 77 ± 5 followed by behavioral testing 30 min later. Mice were euthanized one week later, on approximately PND 84.

Behavioral tests were conducted in the first half of the light phase between 0930 and 1400 in the Behavioral Neuroscience Facility at WSU. All tests were separated by an inter-test interval of at least 48 h and equipment and apparatus were cleaned with 80% ethanol between test animals unless specified otherwise. The testing timeline including treatment is outlined in Figure 1. Behavioral testing order and duration are outlined in Table 1.

### Open field

This test was used to assess locomotion and exploration behaviors relevant to positive symptom domains in schizophrenia (Karl et al., 2007; van den Buuse, 2009) and methods are based on our previous publications (Boucher et al., 2007; Long et al., 2013; Kreilaus et al., 2019; Chesworth et al., 2021). Test mice were placed individually into infrared photobeam controlled test chambers (MED Associates Inc., St Albans, VT, USA) for 30 min. The test arena (43.2 cm × 43.2 cm) was divided into a central and peripheral zone [MED software coordinates for central zone: 3/3, 3/13, 13/3, 13/13 (Karl et al., 2007)] and time and distance in these zones was measured (locomotion defined as two infrared beam breaks within 100 ms). The distance ratio (% distance/time in center) and overall center time were analyzed to assess anxiety-related behaviors.

### Social interaction

This test was used to measure SI behaviors relevant to negative symptom domains in schizophrenia (Wilson and Koenig, 2014) and has been published in our laboratory previously (Long et al., 2012, 2013; Kreilaus et al., 2019; Watt et al., 2020b). The apparatus consisted of a gray Perspex arena (35 × 35 × 30 cm). The paradigm was conducted over two consecutive days. On the first day (habituation) test mice were placed in the arena alone and allowed to explore the apparatus freely for 10 min, then were returned to the home cage. On the following day (test), each test animal was placed into the arena with an age-matched male A/J conspecific in the opposite corner, and mice were allowed to interact freely for 10 min. Frequency of and time spent exerting socio-positive behaviors *sniffing, anogenital sniffing, climbing over/under, and following* were recorded manually using ANY-maze tracking software (Stoelting, Wood Dale, IL, USA). These behaviors were combined to produce a total SI frequency/duration score. Aggressive behaviors of mutant and WT mice were also examined, however, during the study no aggressive behaviors such as *biting* were observed, and therefore were excluded from analysis.

### Prepulse inhibition

This test assessed the acoustic startle response (ASR) and schizophrenia-relevant sensorimotor gating of animals, as previously performed in our laboratory (Long et al., 2006, 2010b). The apparatus consisted of Plexiglas mouse enclosures in startle chambers (SR-Lab, San Diego Instruments, San Diego, CA, USA). The test was conducted over four consecutive days, beginning with three days of habituation to the apparatus and enclosure for 5 min each day with a constant background noise (70 dB). On the fourth day, the 35-min test trial was run and included a 5 min acclimatization period with a 70 dB background noise, followed by 97 trials in a pseudorandomized order: 5 × 70 dB trials (background); 5 × 100 dB trials; 15 × 120 dB trials (startle) and six sets of prepulse-pulse trials using either 74, 82, or 86 dB prepulses presented either 32, 64, 128, or 256 ms [variable interstimulus (prepulse-pulse) interval; ISI] prior to a startle pulse of 120 dB. The intertrial interval (ITI) between individual PPI trials varied randomly from 10 to 20 s. The startle response to each trial was calculated as the mean amplitude detected by the accelerometer. Percentage PPI (% PPI) was calculated as [(mean startle response (120 dB) – PPI response)/mean startle response (120 dB)] × 100%. PPI was averaged across ISI’s to produce a mean %PPI for each prepulse intensity (Chesworth et al., 2021).

### Fear conditioning

This test was used to assess fear-associated learning and memory, which is relevant to cognitive symptom domains in schizophrenia, using methods previously published in our laboratory (Chesworth et al., 2012; Guerra et al., 2021). The apparatus consisted of a FC chamber with a grid floor (MED-VFC-USB-M, Med Associates Inc., St Albans, VT, USA) (29.5 cm × 24.5 cm × 21 cm). FC was run across three days: day (1) conditioning, day (2) context test, and day (3) cue test. During conditioning, mice were placed into the apparatus chamber for 7 min, and after 2 min an 80 dB conditioned stimulus cue was presented for 30 s, co-terminating with a 2 s 0.4 mA foot shock. The tone-shock pairing was repeated 2 min later and the test ended after another 2 min. During conditioning, a vanilla scent cue (Queen™ imitation vanilla essence) was present in the chamber. For the context test (24 h later), mice were returned to the apparatus for 7 min with the vanilla scent cue present. For the cue test 24 h later, mice were returned to the apparatus for 9 min; however, the context of the apparatus was altered with a tent-shape covering around the base grid and no vanilla scent present. After 2 min in the cue test, the tone was played for 5 min, concluding 2 min before the end of the test.

Time spent *freezing* was measured using automated *freezing* detection software, Video Freeze^®^ (Med Associates Inc. – software setting: freezing threshold = 15; detection method = linear; minimum freezing duration = 30 frames). Responses to the cue presentation during the cue test were also analyzed by comparing the percentage of time spent *freezing* in the 2 min prior (i.e., no cue presentation) and the 5 min post cue onset (i.e., during cue presentation).

### Acute Δ^9^-tetrahydrocannabinol challenge

Adolescent and adult *Nrg1* mutants show altered susceptibility to THC (Boucher et al., 2007; Long et al., 2013). Here we examined whether alterations from chronic adolescent CBD could protect against this genetic susceptibility behaviorally. The tests chosen mirrored those used to examine baseline CBD-altered behaviors, however, we did not repeat the FC task as this would be confounded by prior learning in the baseline test. Because CBD may be making alterations to systems that also interact with THC exposure, it was hypothesized that earlier chronic exposure to CBD might impact the effects of an acute dose of THC in mutant animals. Therefore, seven days after the completion of FC, mice were acutely injected with either 3 mg/kg THC or vehicle control, and 30 min later, each mouse was placed into the OF apparatus for 10 min, as described above. Following OF testing, mice were immediately placed into the SI arena with an opponent A/J mouse. After completion of the SI test, mice were placed into the startle chamber apparatus to assess sensorimotor gating. There was a ∼1 min inter-test interval between behavioral tests, which was chosen so that the effects of the THC dose were as consistent as possible between tests, and because 30–120 min post administration is the most relevant for measuring a response to acute THC (Long et al., 2010b). No habituation was required for PPI as this habituation was completed already 1 week prior to the THC battery (Chesworth et al., 2021). These experimental methods are in line with previous studies assessing acute THC effects in this mouse model (Boucher et al., 2007; Long et al., 2010a).

### Tissue collection and western blot

A 1 week delay between behavioral testing and sacrifice was chosen as a washout period from acute THC, so as to not confound effects of CBD with potential alterations from acute THC. One week after the THC test battery, mice were anesthetized with isoflurane gas and perfused with phosphate buffered saline (PBS) transcardially (Watt et al., 2020c). Brains were removed and divided sagittally. The right hemisphere was dissected for the hippocampus, prefrontal cortex, and striatum, which were snap frozen on dry ice upon removal and stored at –80°C. Frozen brain regions (5–20 mg) were homogenized manually with syringes of decreasing needle diameter (21, 25, 27 G) in 12 volumes of radioimmunoprecipitation assay buffer [RIPA; sodium chloride (5 M), Tris-HCl (1 M, pH 8.0), nonidet P-40, sodium deoxycholate (10%), sodium dodecyl sulfate (SDS) (10%), Halt™ Protease and Phosphatase Inhibitor Single-Use Cocktail (100X) and 10 μM PMSF]. Homogenates were centrifuged at 3,750 *g* for 20 min at 4°C and the soluble supernatant was collected. Supernatant was stored at –80°C until used in experiments. Protein content of samples was quantified using Qubit protein assay kit (Life Technologies, Thermofisher Scientific, Carlsbad, CA, USA).

Glutamatergic and GABAergic dysfunction are established pathological characteristics in schizophrenia (Guidotti et al., 2000; Ray et al., 2011; Watt et al., 2020c) and alterations to these neurotransmitter systems are found in *Nrg1* mutants as well (Stefansson et al., 2002; Newell et al., 2013), including increased NMDA receptors in the nucelus accumbens (at 14 but not 20 weeks), decreased NMDA receptors in the thalamus (at 20 but not 14 weeks), and decreased dopamine D2 receptor expression in the striatum (at 14 and 20 weeks) (Newell et al., 2013). The proteins of interest for analysis in this study were: glutamate decarboxylase 67 (GAD67), an enzyme that breaks down excitatory neurotransmitter glutamate into inhibitory neurotransmitter GABA; *N*-methyl-D-aspartate (NMDA) receptor subunit 1 (NR1), the obligatory subunit of the NMDAR; and Alpha-Amino-3-Hydroxy-5-Methyl-4-Isoxazole Propionic Acid (AMPA) receptor obligatory subunit (GluA1). GAD67 has been shown to be decreased in the brains of individuals with schizophrenia (Guidotti et al., 2000; Ray et al., 2011), and reductions in NR1 function have been linked with some symptoms of schizophrenia (Ju and Cui, 2016). GluA1 knockout mice exhibit behavioral deficits relevant to schizophrenia (Barkus et al., 2012, 2014).

Western blotting was conducted on six samples per treatment group (CBD × genotype, *n* = 24) to examine expression of the following protein markers: GAD67 polyclonal antibody (1:2, 000, Thermofisher Scientific [PA5-21397]), AMPA subunit GluA1 [1:10,000, Abcam (ab31232)], NMDAR subunit NR1 [Abcam (ab17345)], and actin housekeeper 1:1,000 [Sigma-Aldrich (A2066)], using methods previously published (Osborne et al., 2019a). Goat anti-rabbit IgG HRP-conjugated secondary antibody [1:10,000, Millipore (AP132P)] and enhanced chemiluminescence was used to detect signals. Signals were quantified using image J software. Data were normalized to actin levels and samples were averaged across duplicates before being analyzed statistically for group differences.

### Statistical analysis

Data for behavioral and molecular analyses were analyzed using two-way or three-way analysis of variance (ANOVA) to investigate main effects and interactions between experimental factors ‘genotype,’ ‘CBD,’ and ‘THC’. Three-way or four-way repeated measures (RM) ANOVAs were used where the within factor was ‘time,’ ‘cue,’ prepulse,’ ‘1-min block,’ or ‘block,’ with the between factors ‘genotype,’ ‘THC,’ and ‘CBD.’ Where interactions were detected, ANOVAs were split by corresponding factor and further ANOVA conducted, as published previously (Long et al., 2012; Cheng et al., 2014). Group differences were regarded as significant if *p* < 0.05, and trends were regarded as *p* = 0.05 – 0.06. *F*-values and degrees of freedom are presented for all ANOVAs and data are shown as means ± standard error of means (SEM). Data normality was assumed as test protocols, facilities, and inbred strains were highly standardized (see methods). These statistical methods have been widely published in our laboratory (Cheng et al., 2014a,b; Coles et al., 2020; Kreilaus et al., 2020; Watt et al., 2020a). Statistical analyses were conducted using SPSS 27 for Mac and GraphPad Prism 8 for Mac.

## Results

### Locomotion, exploration, and anxiety in the open field

**FIGURE 2:**
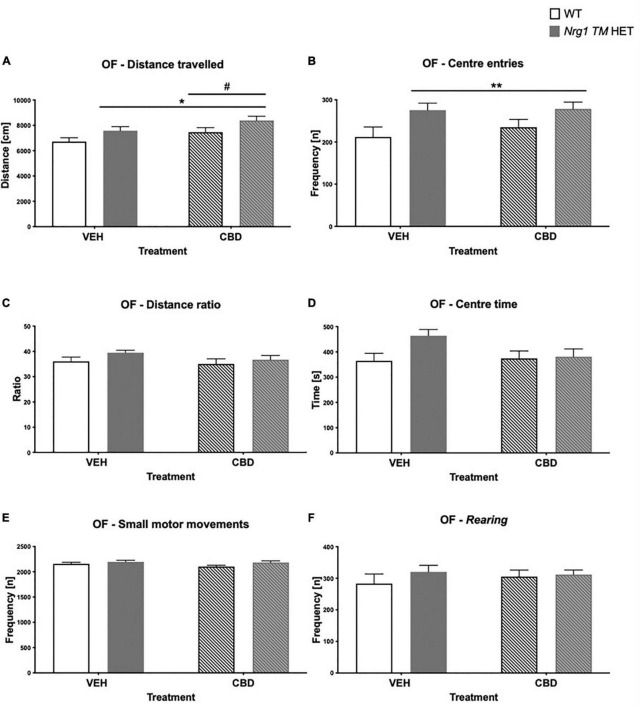
**(A–F)** Open field (OF) – locomotion, exploration, anxiety, and small motor movements after adolescent CBD treatment: Data expressed as mean ± SEM for either wild type-like (WT) or *neuregulin 1 transmembrane domain* heterozygous (*Nrg1 TM* HET) males treated with vehicle (VEH) or cannabidiol (CBD) in adolescence. **(A)** Distance traveled [cm]; **(B)** center entries frequency [n]; **(C)** distance ratio [%]; **(D)** center time [s]; **(E)** small motor movement frequency [n]; and **(F)** *rearing* frequency [n]. Significant two-way ANOVA main effects of CBD are indicated by ^#^*p* < 0.05, and main effects of genotype are shown as *p < 0.05 and **p < 0.01.

There was a main effect of ‘genotype’ for total distance in the OF [two-way ANOVA: ‘genotype’: *F*(1,68) = 6.7; *p* = 0.01; Figure 2A], whereby *Nrg1 TM* HET animals exhibited increased locomotion. Although center entries were higher in *Nrg1* mutant mice [*F*(1,68) = 8.0; *p* = 0.006; Figure 2B], there was no change to other anxiety-like behavior in *Nrg1* mutants [no ‘genotype’ effect for distance ratio, *F*(1,68) = 2.6; *p* = 0.10; Figure 2C; or center time, *F*(1,68) = 3.3*; p* = 0.07; Figure 2D]. *Nrg1* genotype did not affect small motor movements [‘genotype’ *F*(1,68) = 3.6; *p* = 0.063; Figure 2E] or *rearing* frequency [*F*(1,68) = 0.1; *p* = 0.30; Figure 2F].

There was a main effect of ‘CBD’ where CBD increased total distance traveled across both genotypes [*F*(1,68) = 5.07; *p* = 0.03; Figure 2A]. There were no ‘CBD’ × ‘genotype’ interactions for any behavior (all *p’s* > 0.05).

### Social behaviors

**FIGURE 3:**
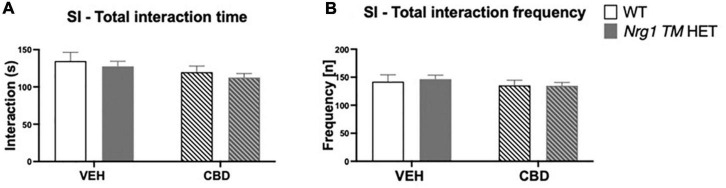
**(A,B)** Social interaction (SI) after adolescent CBD treatment: Total interaction time [s] and frequency [n] expressed as mean ± SEM for either wild type-like (WT) or *neuregulin 1 transmembrane domain* heterozygous (*Nrg1 TM* HET) mice treated during adolescence with either vehicle (VEH) or cannabidiol (CBD); **(A)** total social interaction time [s]; **(B)** total social interaction frequency [n].

No ‘genotype’ two-way ANOVA main effects were detected for total SI time or frequency, or the duration or frequency of individual socio-positive behaviors (Table 2, all *p’s* > 0.05). There was no significant main effect of ‘CBD’ on total SI time [*F*(1,68) = 3.6; *p* = 0.067; Figure 3A] or total SI frequency [*F*(1,68) = 1.2; *p* = 0.30; Figure 3B]. Furthermore, *nosing* time [*F*(1,68) = 3.4; *p* = 0.07; Table 2], *climbing on/over* time [*F*(1,68) = 3.2; *p* = 0.076; Table 2], and *climbing on/over* frequency [*F*(1,68) = 3.5; *p* = 0.07; Table 2] were also unaffected by chronic CBD. Finally, there were no interactions between ‘genotype’ and ‘CBD’ for socio-positive behaviors (all *p’s* > 0.05).

**TABLE 2:** Socio-positive behaviors after adolescent CBD treatment: Duration [s] and frequency [n] of *nosing*, *anogenital sniffing*, *climbing on/over*, and *following* the A/J mouse in the social interaction (SI) test.

Genotype	WT		*Nrg1 TM* HET	
Chronic treatment	VEH	CBD	VEH	CBD
*Nosing [s]*	84.7 ± 5.6	77.9 ± 4.7	79.6 ± 3.8	71.2 ± 2.7
*Anogenital sniffing [s]*	30.2 ± 4	27.2 ± 2.8	30.3 ± 2.6	26.8 ± 2
*Climbing On/Over [s]*	15.1 ± 3	12 ± 2.1	13.3 ± 1.7	9.3 ± 0.9
*Following [s]*	4.7 ± 1.3	3.3 ± 0.9	4.3 ± 0.9	5.2 ± 0.9
*Nosing [n]*	87.6 ± 5.8	86.7 ± 5.3	91.3 ± 4	85.1 ± 3.2
*Anogenital sniffing [n]*	29.8 ± 3.1	29 ± 2.3	32 ± 2.1	30.8 ± 1.6
*Climbing On/Over [n]*	17.1 ± 3	14.5 ± 2.3	16.2 ± 1.6	11.6 ± 1
*Following [n]*	7.6 ± 2.2	5.2 ± 1.3	6.9 ± 1.2	7.2 ± 1

Data expressed as mean ± SEM for wild type-like (WT) and neuregulin 1 transmembrane domain heterozygous (Nrg1 TM HET) mice treated during adolescence with either vehicle (VEH) or cannabidiol (CBD).

### Prepulse inhibition

**FIGURE 4:**
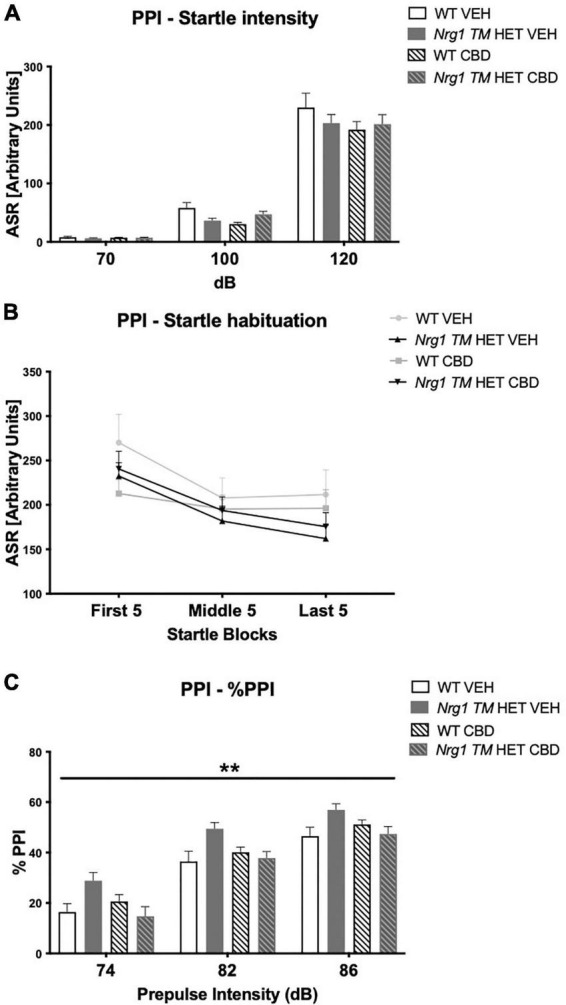
**(A–C)** Acoustic startle response and prepulse inhibition (PPI) after adolescent CBD treatment: **(A)** average startle [ASR], **(B)** average first and last five startle responses (startle habituation), and **(C)** percentage PPI (% PPI) across a 74, 82, and 86 dB prepulse intensities, expressed as mean ± SEM for wild type-like (WT) and *neuregulin 1 transmembrane domain* heterozygous (*Nrg1 TM* HET) mice treated with adolescent vehicle (VEH) or cannabidiol (CBD). Main effects of ‘genotype’ indicated by ** *p* < 0.01.

*Nrg1 TM* HET animals had significantly higher %PPI across prepulse intensities compared to WT controls [*F*(1,68) = 8.5; *p* = 0.005; Figure 4C], but this was unaffected by CBD (*p* > 0.05). Three-way RM ANOVA revealed that neither ‘genotype’ [*F*(3,68) = 1.9; *p* = 0.40; Figure 4A] nor CBD treatment [*F*(1,68) = 3.6; *p* = 0.063; Figure 4A] had an overall effect on average startle across startle intensities. Furthermore, no interactions between ‘genotype,’ ‘CBD,’ or ‘startle pulse intensity’ were present (all *p’s* > 0.05). As expected, higher startle pulse intensities elicited greater startle response [three-way RM ANOVA for ‘startle pulse intensity’: *F*(2,136) = 496.9; *p* < 0.0001; Figure 4A]. Startle habituation was present in all animals regardless of ‘genotype’ or ‘CBD’ [three-way RM ANOVA for ‘block’: *F*(2,136) = 25.2; *p* < 0.0001; Figure 4B – no interactions with ‘genotype’ and/or ‘CBD’: all *p’s* > 0.05].

As expected, %PPI increased with increasing prepulse intensities [three-way RM ANOVA for ‘prepulse’: *F*(2,136) = 501.7; *p* < 0.0001; Figure 4C].

### Fear conditioning

**FIGURE 5:**
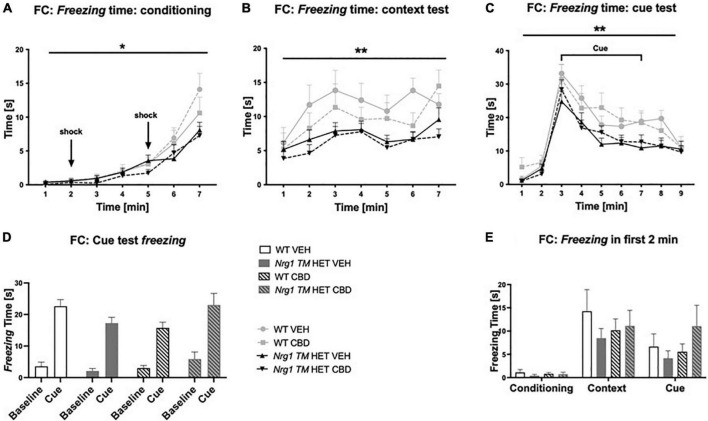
**(A–E)** Time spent *freezing* in fear conditioning (FC) after adolescent CBD treatment: Duration [s] of *freezing* expressed as mean ± SEM for either wild type-like (WT) or *neuregulin 1 transmembrane domain* heterozygous (*Nrg1 TM* HET) mice treated with vehicle (VEH) or cannabidiol (CBD) in adolescence. **(A)** *Freezing* time across conditioning; **(B)** *freezing* time across context test; **(C)** *freezing* time across cue test; **(D)** average baseline *freezing* vs. during the cue; and **(E)** baseline *freezing* across test days. Genotype main effects indicated by * *p* < 0.05 and ** p < 0.01.

Conditioning: *Freezing* at baseline in the first two minutes of conditioning was unaffected by ‘genotype’ [*F*(1,68) = 0.6; *p* = 0.40] or ‘CBD’ treatment [*F*(1,68) = 0.9; *p* = 0.30]. *Freezing* during the full 7-min conditioning session increased across 1-min blocks [three-way RM ANOVA for ‘time’: *F*(6,408) = 71.9; *p* < 0.0001] and *Nrg1 TM* HET mice *froze* less than WT mice [‘genotype’ *F*(1,68) = 4.13; *p* = 0.05; Figure 5A]; this was not altered by CBD treatment (no ‘CBD’ main effect or interactions, all *p*’s > 0.05). An overall ‘time’ × ‘genotype’ interaction was detected [*F*(6,408) = 4.18; *p* = 0.0004] but when split by ‘genotype,’ both *Nrg1 TM* HET animals [*F*(6,246) = 33.9; *p* < 0.0001] and WT animals [*F*(6,162) = 34.1; *p* < 0.0001] increased *freezing* across the conditioning period.

#### Context test

Three-way RM ANOVA revealed a main effect of ‘genotype’ for *freezing* in the context test, with overall *freezing* being reduced in *Nrg1 TM* HET animals compared to WTs [*F*(1,68) = 9.8; *p* = 0.003; Figure 5B]. No interactions with ‘time’ or ‘CBD’ were evident (all *p’s* > 0.05), suggesting that reduced *freezing* in *Nrg1* mutants was apparent throughout the context test and was unaffected by CBD treatment.

#### Cue test

*Freezing* behavior changed across the course of the test [‘time’ *F*(8,536) = 66.2; *p* < 0.0001] and again, *Nrg1 TM* HET animals *froze* less overall than their WT counterparts [‘genotype’ *F*(1,67) = 7.9; *p* = 0.006; Figure 5C]. There were no interactions between ‘time’ and ‘CBD’ or ‘genotype,’ and no main effects of ‘CBD’ (all *p’s* > 0.05). Comparing average *freezing* before cue presentation with during cue presentation, all mice displayed increased *freezing* to the cue [three-way RM ANOVA for ‘cue’: *F*(1,67) = 263.1; *p* < 0.0001] regardless of treatment condition or genotype (i.e., no interactions with ‘cue’: all *p’s* > 0.05; Figure 5D). When *freezing* was analyzed during the cue presentation only, we detected reduced *freezing* in *Nrg1* mutant animals [two-way ANOVA main effect of ‘genotype’: *F*(1,67) = 7; *p* = 0.01; data not shown].

To check that all mice formed an association between the tone, context and shock, *freezing* in the first 2 min was compared across the three experimental days. *Freezing* in the first 2 min of the context and cue tests was significantly higher than during the first 2 min of conditioning [three-way RM ANOVA for ‘days’: *F*(2,136) = 30.3; *p* < 0.0001; Figure 5E]. This indicates all mice remembered the tone-context-shock association (no ‘genotype’ or ‘CBD’ effects or interactions with ‘days’ were present; all *p’s* > 0.05).

### Locomotion, exploration, and anxiety after acute Δ^9^-tetrahydrocannabinol

**FIGURE 6:**
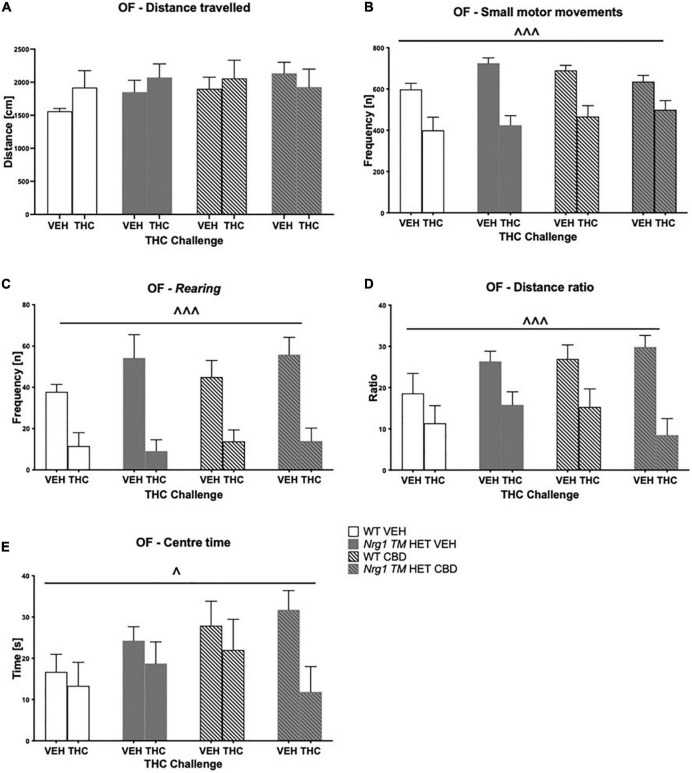
**(A–E)** Open field (OF) – locomotion, exploration, and small motor movements after chronic CBD treatment and acute THC challenge: Data expressed as mean ± SEM for either wild type-like (WT) or *neuregulin 1 transmembrane domain* heterozygous (*Nrg1 TM* HET) mice treated with vehicle (VEH) or cannabidiol (CBD). Mice were acutely challenged with vehicle or Δ^9^-tetrahydrocannabinol (THC) 30 min prior to testing. **(A)** Distance traveled [cm]; **(B)** small motor movement frequency [n]; **(C)** *rearing* frequency [n]; **(D)** distance ratio; **(E)** center time [s]. Significant three-way ANOVA main effects of ‘THC’ are shown as ^∧^ *p* < 0.05 and ^∧^ ^∧^ ^∧^ *p* < 0.001.

Hyperactivity in the OF in *Nrg1* mutants was not present in this test. There were no effects of acute THC, or CBD or genotype on total distance traveled in the OF (three-way ANOVA main effects ‘THC,’ ‘genotype’ and ‘CBD’; all *p’s* > 0.05, no interactions; Figure 6A). THC significantly decreased small motor movement frequency [*F*(1,64) = 50.5; *p* < 0.0001; Figure 6B] and *rearing* frequency [*F*(1,64) = 43.4; *p* < 0.0001; Figure 6C] across groups; however, effects of acute THC challenge did not vary with chronic CBD treatment or *Nrg1* genotype (no interactions: all *p’s* > 0.05). Acute THC also decreased distance ratio [*F*(1,64) = 5.8; *p* < 0.0001; Figure 6D] and time spent in the center of the OF compared to VEH controls [*F*(1,64) = 4.9; *p* = 0.03; Figure 6E]. There were no main effects of ‘genotype’ or ‘CBD’ and no significant interactions for any other parameters measured in the OF (all *p’s* > 0.05).

### Social behaviors after acute Δ^9^-tetrahydrocannabinol

**FIGURE 7:**
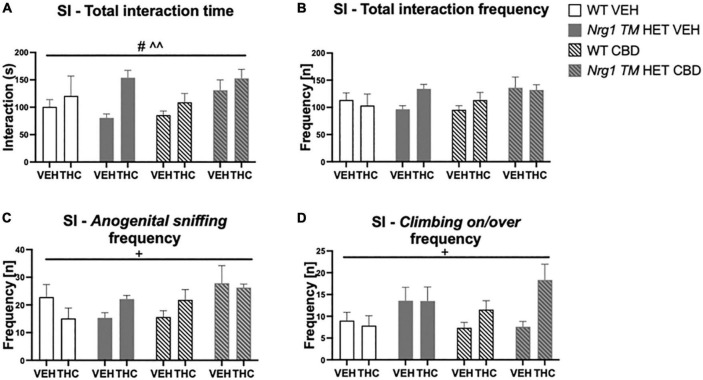
**(A–D)** Social interaction (SI) after chronic CBD treatment and acute THC challenge: Total interaction time [s] and frequency [n] expressed as mean ± SEM for either wild type-like (WT) or *neuregulin 1 transmembrane domain* heterozygous (*Nrg1 TM* HET) mice treated with vehicle (VEH) or cannabidiol (CBD), then later vehicle or Δ^9^-tetrahydrocannabinol (THC). **(A)** Total interaction time [s]; **(B)** total interaction frequency [n]; **(C)** anogenital sniffing frequency [n]; and **(D)** climbing on/over frequency [n]. Significant effects of ‘CBD’ are indicated by ^#^ *p* < 0.05, and significant effects of ‘THC’ are indicated by ^^*p* < 0.01. Significant interactions between factors are indicated by +*p* < 0.05.

Acute low dose THC increased total SI time [*F*(1,64) = 8.3; *p* = 0.005; Figure 7A], as did chronic CBD treatment [*F*(1,64) = 4.5; *p* = 0.04; Figure 7A; no interactions]. Total SI frequency also tended to be increased by CBD treatment [*F*(1,64) = 3.8; *p* = 0.054]. There were no interactions between any factors (all *p’s* > 0.05; Figure 7B).

Analyzing individual SI behaviors, THC increased *nosing* time [*F*(1,64) = 7.6; *p* = 0.007; Table 3], but no significant effects of ‘CBD’ or ‘genotype’ and no interactions were present for *nosing* time or *nosing* frequency (all *p’s* > 0.05). THC did not affect duration or frequency of *anogenital sniffing* (all *p’s* > 0.05; Table 3) whereas CBD increased *anogenital sniffing* frequency [*F*(1,64) = 6.2; *p* = 0.01; Table 3]. Interestingly, an interaction between ‘THC’ and ‘genotype’ was also evident for *anogenital sniffing* frequency [*F*(1,64) = 5.3; *p* = 0.02; Figure 7C]. When split by ‘THC,’ an effect of ‘genotype’ was only found in VEH-treated animals [*F*(1,34) = 7.4; *p* = 0.01] but not THC-treated animals [*F*(1,36) = 0.2; *p* = 0.70], indicating that *Nrg1 TM* HET VEH animals showed less *anogenital sniffing* than WT VEH mice, but not after THC challenge. When split by ‘genotype,’ an effect of ‘THC’ was found in *Nrg1 TM* HET animals [*F*(1,43) = 5.5; *p* = 0.02] but not WT animals [*F*(1,29) = 1.2; *p* = 0.30], demonstrating increased *anogenital sniffing* in *Nrg1 TM* HET mice following THC exposure, and confirming that mutant mice appear to be more susceptible some THC behavioral effects. These findings were unaffected by CBD treatment (all *p’s* > 0.05 for interactions with CBD). *Nrg1 TM* HET animals also spent less time engaged in *anogenital sniffing* [*F*(1,64) = 4.0; *p* = 0.049; Table 3] regardless of CBD treatment condition (all *p’s* > 0.05).

**TABLE 3:** Socio-positive behaviors after chronic CBD treatment and acute THC challenge: Duration [s] and frequency (fq) [n] of *nosing*, *anogenital sniffing*, *climbing on/over*, and *following* the A/J mouse in the social interaction (SI) test following acute THC exposure.

Genotype	WT				*Nrg1 TM* HET			
Chronic treatment	VEH		CBD		VEH		CBD	
THC challenge	VEH	THC	VEH	THC	VEH	THC	VEH	THC
*Nosing [s]^^*	68.9 ± 8.5	90.4 ± 27.4	85.8 ± 10.6	95.4 ± 13.8	59.3 ± 5.15	73.4 ± 10.7	59.2 ± 5.2	107.3 ± 8.9
Anogenital Sniffing [s]*	20.9 ± 5.9	17.5 ± 5.9	28 ± 6.7	33.7 ± 4.2	15.5 ± 2.6	21.6 ± 4.7	13.6 ± 2.3	24.1 ± 3.6
*Climbing On/Over [s]^#*	7.9 ± 1.4	8.23 ± 3	13.3 ± 3.8	16.1 ± 5.6	7.7 ± 1.5	10.8 ± 2.1	6.2 ± 1.34	20.2 ± 5.4
*Following [s]*	3.2 ± 1.5	4.61 ± 3.3	3.9 ± 1.2	7.4 ± 1.9	3.1 ± 1.4	3.1 ± 1.7	1.5 ± 0.6	2.2 ± 1.3
*Nosing [n]*	77.8 ± 7.5	75.3 ± 14.1	88.8 ± 10.7	84 ± 6.9	68.4 ± 5.7	76.5 ± 8.6	71.5 ± 4.5	91 ± 5.3
*Anogenital Sniffing [n]* +##	22.8 ± 4.5	15.1 ± 3.7	27.8 ± 6.3	26.2 ± 1.3	15.6 ± 2.3	21.8 ± 3.7	15.3 ± 1.9*	22.1 ± 1.3
*Climbing On/Over [n]* +	9 ± 1.9	7.85 ± 2.3	13.6 ± 3.1	13.5 ± 3.2	7.4 ± 1.2	11.5 ± 2	7.6 ± 1.2**	18.3 ± 3.6
*Following [n]*	4 ± 1.7	5.1 ± 3.7	6 ± 1.7	8.4 ± 1.7	4.2 ± 1.8	3.7 ± 1.5	2.1 ± 0.6	2.5 ± 1.1

Data expressed as mean ± SEM for wild type-like (WT) and neuregulin 1 transmembrane domain heterozygous (Nrg1 TM HET) mice treated with vehicle (VEH) or cannabidiol (CBD) then later vehicle or Δ^9^-tetrahydrocannabinol (THC). Main effects are indicated with: ‘genotype’ *p < 0.05; ‘CBD’ ^#^*p* < 0.05, ^##^*p* < 0.01; and ‘THC’ ^*p* < 0.05, ^^*p* < 0.01. Interactions between ‘THC’ and ‘genotype’ were found for anogenital sniffing [+*p* = 0.02] and climbing on/over [+*p* = 0.02] frequency. Split by effects of ‘genotype’ within these interactions are indicated with * *p* < 0.05 and ** *p* < 0.01.

Δ^9^-tetrahydrocannabinol challenge increased *climbing on/over* [time spent: *F*(1,64) = 4.7; *p* = 0.03 – trend for frequency: *F*(1,64) = 3.9; *p* = 0.051; Table 3 and Figure 7D] and a ‘THC’ by ‘genotype’ interaction was also evident [frequency: *F*(1,64) = 5.5; *p* = 0.02; Table 3]. When split by ‘THC,’ only *Nrg1* mice of the VEH group showed a reduced *climbing on/over* frequency [*F*(1,34) = 4.4; *p* = 0.04] but not the THC-treated group [*F*(1,36) = 4.6; *p* = 0.40]. When split by ‘genotype’ instead, acute THC challenge increased this behavior in *Nrg1 TM* HET animals only [*F*(1,43) = 12; *p* = 0.001] but not WT animals [*F*(1,29) = 0.05; *p* = 0.80], again suggesting that *Nrg1* mutant mice are more susceptible to some of the behavioral effects of THC. CBD treatment also increased the time spent *climbing on/over* [*F*(1,64) = 5.09; *p* = 0.03; Table 3] but had no other effects.

Finally, neither THC challenge, nor *Nrg1* genotype or adolescent CBD affected *following* behavior (all *p’s* > 0.05, no interactions, Table 3).

### Prepulse inhibition after acute Δ^9^-tetrahydrocannabinol

**FIGURE 8:**
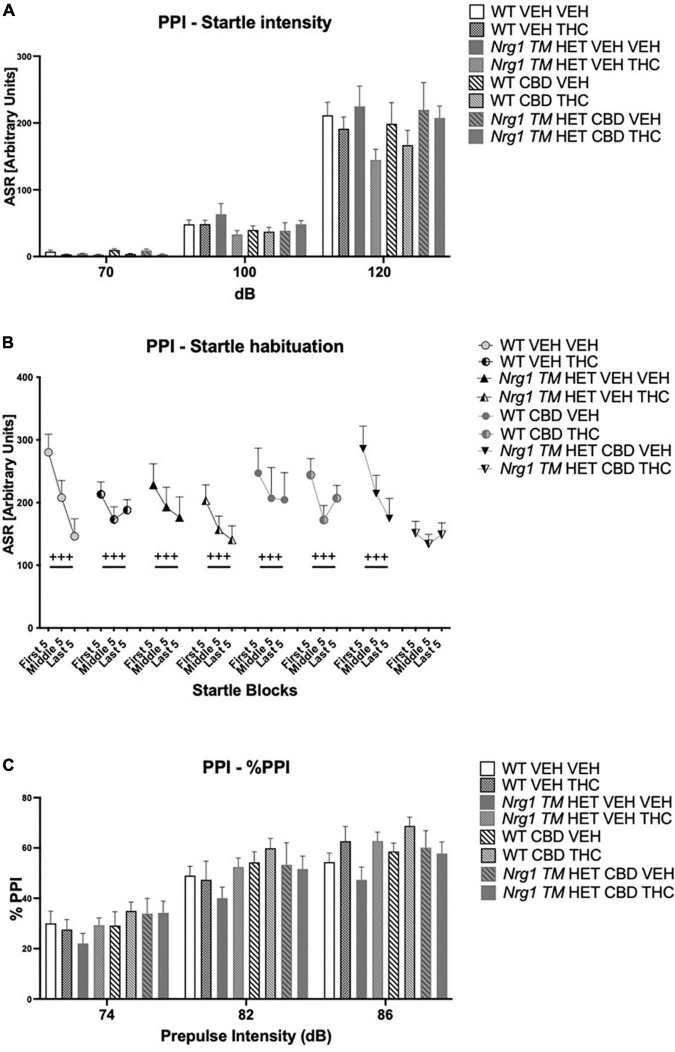
**(A–C)** Acoustic startle response and prepulse inhibition (PPI) after chronic CBD treatment and acute THC challenge: **(A)** average startle [ASR], **(B)** average first and last five startle responses (startle habituation), and **(C)** percentage PPI (% PPI) using 74, 82, and 86 dB prepulses expressed as mean ± SEM for either wild type-like (WT) or *neuregulin 1 transmembrane domain* heterozygous (*Nrg1 TM* HET) mice treated with vehicle (VEH) or cannabidiol (CBD), then later vehicle or Δ^9^-tetrahydrocannabinol (THC). RM effects showed animals startled more at a higher pulse, had higher PPI at a higher pulse, and habituated through the test to startle pulses (*p’s* < 0.001).

Acoustic startle increased with higher startle pulse intensities [four-way RM ANOVA for ‘startle pulse intensity’: *F*(2,128) = 364.6; *p* ≤ 0.0001; Figure 8A]. There was also a trend for ‘THC’ to decrease the startle response compared to VEH-treated animals [*F*(1,64) = 3.7; *p* = 0.058; Figure 8A; no interactions with ‘CBD’ or ‘genotype’ and no other main effects].

All animals habituated to the startle stimulus [‘startle block’: *F*(2,128) = 37.1; *p* < 0.0001; Figure 8B]. An interaction between ‘startle block,’ ‘genotype,’ ‘CBD,’ and ‘THC’ [*F*(2,128) = 7.3; *p* = 0.003] was also present. To further investigate this, data were split by all between factors (‘genotype,’ ‘CBD,’ and ‘THC’) to investigate the RM effect of ‘startle block’ in each individual group. While every other group habituated (all *p’s* < 0.05), *Nrg1 TM* HET mice treated with chronic CBD and then acutely challenged with THC exhibited no significant RM effect of ‘startle block’ [*F*(2,16) = 1.1; *p* = 0.30] suggesting habituation was impaired in these mice (Figure 8B).

%PPI increased with increasing prepulse intensities [four-way RM ANOVA for ‘prepulse’: *F*(2,64) = 371.7; *p* ≤ 0.0001; Figure 8C]. No main effects of ‘THC,’ ‘genotype,’ or ‘CBD’ were found for %PPI (all *p’s* > 0.05) but there was an interaction between ‘prepulse’ and ‘THC’ [*F*(2,64) = 3.1; *p* = 0.049]. However, when split by ‘THC’ no further main effects or interactions were present (all *p’s* > 0.05), and both THC-treated [‘prepulse’: *F*(2,66) = 208.7; *p* < 0.0001] and VEH-treated groups [‘prepulse’: *F*(2,62) = 165.7; *p* < 0.0001] showed increasing PPI with higher prepulse intensities.

### Western blotting

**FIGURE 9:**
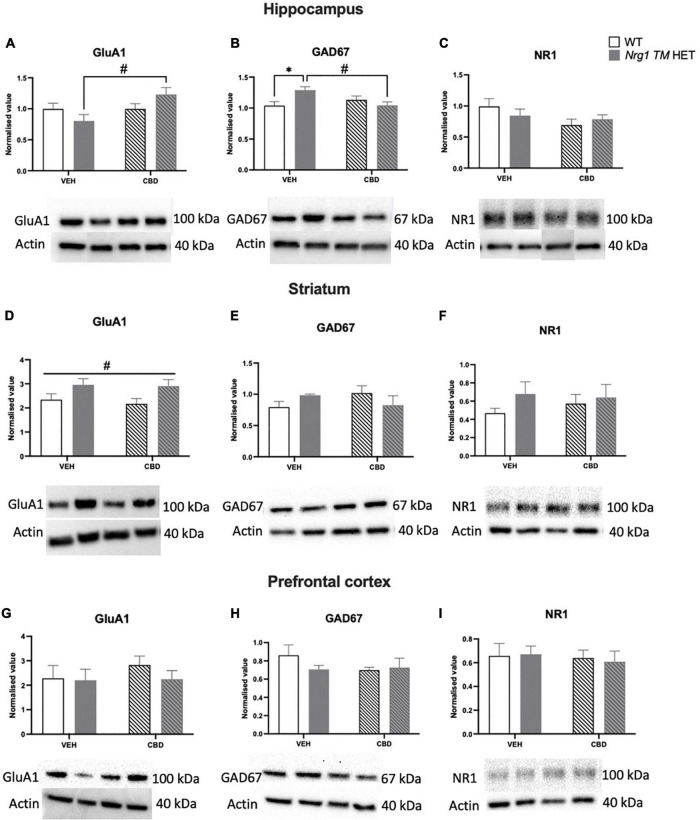
**(A–I)** Glutamatergic and GABA-related protein levels in the hippocampus **(A–C)**, striatum **(D–F)**, and prefrontal cortex (PFC) **(G–I)**: Glutamate AMPA receptor subunit GluA1, glutamate decarboxylase 67 (GAD67), and NMDAR1 subunit NR1 protein levels expressed as mean ± SEM for either wild type-like (WT) or *neuregulin 1 transmembrane domain* heterozygous (*Nrg1 TM* HET) mice treated with vehicle (VEH) or cannabidiol (CBD). Split comparisons indicated with ‘genotype’ split effect *p < 0.05, and ‘treatment’ split effect ^#^*p* < 0.05. Main ‘treatment’ effect in **(D)** indicated with ^#^*p* < 0.05. Western blotting bands are in order of the groups represented in the graphs. Bands in the hippocampus have been assembled post-experiment in this order as were run in reverse order, however were not altered otherwise.

A two-way ANOVA revealed a main effect of ‘CBD’ but not ‘genotype’ (*p* > 0.05), whereby CBD treatment increased GluA1 protein levels in the hippocampus [*F*(1,20) = 4.7; *p* = 0.04]. A significant ‘genotype’ × ‘CBD’ interaction was also observed [*F*(1,20) = 4.7; *p* = 0.04; Figure 9A]. Indeed, when split by genotype, hippocampal GluA1 expression was significantly increased by CBD treatment in *Nrg1* mutants compared to VEH-treated mutant mice [*F*(1,12) = 7.9; *p* = 0.02; Figure 9A]; however, this increase was not found in WT mice [*F*(1,12) = 0.0001; *p* = 0.99]. When split by CBD, no genotype effects were observed (all *p’s* > 0.05).

No main effects of ‘genotype’ or ‘CBD’ were evident for hippocampal GAD67 levels (all *p*’s > 0.05). However, a ‘genotype’ × ‘CBD’ interaction was present [*F*(1,20) = 8.03; *p* = 0.01; Figure 9B], which when split by ‘CBD’ showed increased GAD67 in *Nrg1* VEH compared to WT VEH mice [*F*(1,12) = 8.4; *p* = 0.02]. When split by ‘genotype,’ CBD treatment decreased GAD67 in *Nrg1 TM* HET males (vs. *Nrg1* VEH), but not WT mice [*F*(1,12) = 9.7; *p* = 0.01].

NR1 protein levels were unchanged by genotype or CBD treatment in the hippocampus (all *p*’s < 0.05; Figure 9C).

In the striatum, GluA1 was higher in *Nrg1* mutants compared to WT animals regardless of CBD treatment [‘genotype’ main effect: *F*(1,20) = 7.6; *p* = 0.01; Figure 9D]. CBD treatment did not affect GluA1 and there was no interaction with *Nrg1* genotype (all *p’s* < 0.05).

GAD67 and NR1 protein levels in the striatum were unaltered by either genotype or treatment, with no interaction (all *p’s* < 0.05; Figures 9E,F).

In the PFC, there was no impact of ‘CBD’ or ‘genotype’ and no interactions for any proteins investigated (all *p’s* < 0.05; Figures 9G–I).

## Discussion

Here we found that, overall, CBD did not reverse schizophrenia-relevant behaviors in *Nrg1* mutant mice. CBD increased locomotion at baseline and increased social behaviors under THC in all mice without affecting SI at baseline. Acute THC increased anxiety-like behavior and decreased exploration and fine motor movements in the OF, and increased social behaviors. CBD and THC combined impaired startle habituation specifically in *Nrg1* mutants. CBD affected some glutamatergic and GABAergic markers in *Nrg1* mice, where CBD increased GluA1 and decreased GAD67 levels in *Nrg1* mutant mice but not in WT controls.

Cannabidiol did not reduce the expression of schizophrenia-relevant behaviors in *Nrg1 TM* HET mice. *Nrg1* mice showed schizophrenia-relevant behaviors including hyperlocomotion, some reduced social behaviors, and impaired fear-associated memory recall (reported previously in Karl et al., 2007; Duffy et al., 2010), but this was unaffected by chronic adolescent CBD treatment. Contrary to our findings, previous work shows chronic adolescent CBD prevented poly I:C-induced cognitive deficits and social withdrawal in rats and mice (Osborne et al., 2017, 2019a; da Silva et al., 2020). Differences between these studies and ours include the model system used, i.e., gene mutation versus neonatal poly I:C administration, which may impact the effectiveness of CBD as a treatment candidate. For example, CBD is anti-inflammatory (Burstein, 2015) and in poly I:C rats, there can be significant and sustained neuroinflammation in adult offspring (Ding et al., 2019), whereas neuroinflammation is less pronounced in *Nrg1 TM* HET mice, with only small changes to serum cytokine levels are evident in adult *Nrg1* male mutants (Desbonnet et al., 2012, 2017). Thus, CBD may be effective in model systems and individuals with schizophrenia with high levels of neuroinflammation (Fillman et al., 2013, 2016; North et al., 2021), but may be less effective when schizophrenia-induced behaviors are less dependent on neuroinflammation.

The current study did not find social deficits present in adolescence in male *Nrg1* mutant animals in baseline testing. While this is the case, social deficits were present some weeks later under acute THC treatment, suggesting deficits of social behavior may develop later in this model, after the adolescent period. This is supported by a previous treatment study that did not find social deficits in male adolescent *Nrg1* mutants (Long et al., 2013).

Cannabinoids such as THC and CBD have differential effects on *Nrg1* mutants during adolescence and adulthood (Arnold et al., 2007; Boucher et al., 2007; Long et al., 2013), and while chronic CBD has been assessed previously in adult male mutants (Long et al., 2012), it is not known whether chronic CBD in adolescence could be different. Due to its key role in neurodevelopment, adolescence may provide a window for utilizing CBD’s protective effects as a preventative. Adolescent CBD induced a mild hyperlocomotive phenotype in the first OF test in all animals, regardless of genotype. This is the first investigation of chronic adolescent CBD on locomotor activity. Interestingly, our findings contrast with data from chronic CBD in adult animals, where CBD did not induce hyperlocomotion itself (Moreira and Guimarães, 2005; Long et al., 2010b,^2012^; Gururajan et al., 2012) or even reduced locomotion in adult C57BL/6 mice (Schleicher et al., 2019). It is possible that this effect of CBD is only relevant to adolescent CBD exposure as we did not detect increased locomotor activity in CBD-treated mice under acute THC which was conducted in later adolescence, suggesting pro-locomotor effects of CBD may be highly age-dependent.

CBD had different effects on social behavior depending on treatment duration: CBD increased social behaviors in both genotypes but only when given longer-term (i.e., 6 weeks *versus* 3 weeks). This is the first time CBD has been found to increase social behaviors in *control* animals. Previous work found similar effects only in rodent models of schizophrenia and Dravet syndrome (Long et al., 2012; Osborne et al., 2017; Patra et al., 2020), and one study actually reported adolescent CBD decreasing social behaviors in control female rats (Osborne et al., 2019a). It is possible that CBD treatment takes longer than three weeks to improve social behaviors in control animals, and this may be due to sensitization of the serotonergic effects of CBD. There is evidence for sensitization of the serotonergic system with chronic CBD, as chronic, but not acute CBD increases serotonin release in the ventromedial PFC (Linge et al., 2016). Importantly, prosocial behavior is associated with higher serotonin levels (Dölen et al., 2013), and brain regions including the PFC mediate social behaviors (Ko, 2017). CBD is a 5-HT1A agonist and prosocial effects of CBD can be mediated by 5-HT1A receptors (Hartmann et al., 2019). Thus, it is possible that prosocial effects of chronic CBD may be due to a sensitized serotonergic response (e.g., increased serotonin release, potential upregulation of 5-HT1A receptor expression) which only occurs after extended CBD administration. As this was the first study to investigate how > 3 weeks of CBD treatment affects social behavior, future studies should consider how extended CBD treatment durations can affect prosocial behaviors.

Our study is the first to determine that adolescent CBD does not affect sensorimotor gating in *Nrg1 TM* HET mice. Effects of CBD in adult animals appear dependent on treatment duration, as acute CBD robustly reverses PPI deficits of mice after acute MK-801 or amphetamine challenge (Long et al., 2006; Pedrazzi et al., 2015, 2021), but chronic CBD is less effective and only reverses PPI deficits at one prepulse intensity in a chronic MK-801 mouse model (Gomes et al., 2015). Interestingly, PPI was elevated in *Nrg1* mutant mice in the first PPI test (i.e., baseline), but not the second PPI test (i.e., during the THC battery). This is opposite to previous findings of reduced PPI in *Nrg1 TM* HET male mice (Stefansson et al., 2002; Boucher et al., 2007, 2011; Desbonnet et al., 2012; Long et al., 2013) but some studies have reported elevated PPI in adult *Nrg1 TM* HET male (Karl et al., 2011) and female mice (Long et al., 2010a) in the past. This phenotype variability is in line with earlier (van den Buuse et al., 2009; Karl et al., 2011) and late adolescence (Long et al., 2013), and PPI deficits can disappear with repeated testing (Boucher et al., 2011).

Long-term oral CBD treatment reduced *freezing* in the cue test of all females regardless of genotype. While it is well-established that acute systemic CBD can impair fear memory consolidation (Stern et al., 2018; Shallcross et al., 2019; Han et al., 2022), including in female mice (Montoya et al., 2020), effects of chronic CBD on fear memory have had limited investigation and chronic CBD does not appear to affect fear memory acquisition (Cheng et al., 2014, 2014c). Considering CBD-induced differences in *freezing* were very limited in this study, future research should consider evaluating the effects of long-term CBD on fear learning in more detail.

We replicated sedative and anxiogenic-like effects of THC in the OF, and increased sensitivity to acute THC in *Nrg1 TM* HET mice. In the OF, acute THC increased anxiety-like behavior and decreased exploration across genotypes and treatment groups, similar to previous work (Schramm-Sapyta et al., 2007; Long et al., 2010b; Schreiber et al., 2019). Importantly, we extend prior research to demonstrate that anxiogenic and exploration-inhibiting effects of THC can be found at lower doses than previously reported i.e., 3 mg/kg (Long et al., 2010b). Interestingly, THC increased social behavior overall, where higher doses, e.g., 5 mg/kg have been shown to decrease social behavior (Arnold et al., 2007), and this effect was more pronounced in *Nrg1* mutants, similar to our previous findings of increased THC susceptibility of *Nrg1* mutant mice (Boucher et al., 2007). While acute THC increasing social behaviors does not reflect clinical research (Haney et al., 1999), adolescent *Nrg1* mutants have been shown to be protected against a reduction in social behaviors caused by chronic 10 mg/kg THC where WT mice were not (Long et al., 2013). It is possible that a lower acute dose of THC may increase social behaviors selectively in *Nrg1* mutants. Acute THC increasing social behaviors is however a novel finding, and requires replication and further investigation to understand the mechanisms driving this finding (e.g., if it is related to anxiety-like behavior), as one recent study has found a low dose (3.2 and 6.4 mg/kg) of THC can decrease anxiety measures in the elevated plus maze (Liu et al., 2022). Certainly, an increase in SI has been linked to decreased anxiety in previous work (File and Hyde, 1978), and this could be the mechanism by which this is occurring in the current study.

Few changes in THC sensitivity by chronic CBD were found in this study. Indeed, the only change detected was impaired startle habituation in *Nrg1* mutants treated with both CBD and THC. We found increased hippocampal GluA1 levels in *Nrg1* mutants following adolescent CBD, suggesting increased hippocampal excitability. Intra-hippocampal NMDA receptor antagonist infusions disrupt PPI in rats (Shoemaker et al., 2005), and GluA1 receptors are necessary for short-term habituation to recently experienced stimuli (Sanderson et al., 2010; Sanderson and Bannerman, 2012), such as a startle pulse, suggesting a role for hippocampal glutamatergic receptors, and possibly GluA1 receptors in regulating startle habituation. As we did not see CBD-induced changes to startle or PPI in *Nrg1 TM* HET mice, it is possible that CBD had subthreshold effects on sensorimotor gating, and a combination of CBD and THC was needed to exacerbate glutamatergic receptor imbalance in *Nrg1 TM* HET mice thereby impairing startle habituation. Indeed, in cannabis users, the combination of acute THC and CBD reduces mismatch negativity (an endophenotype for schizophrenia) more than acute CBD or THC alone (Greenwood et al., 2022). Considering THC can increase hippocampal GluA1 receptor levels in rats (Rubino et al., 2015; Zamberletti et al., 2016), it is possible THC could have exacerbated effects of CBD on startle habituation in *Nrg1 TM* HET mice, via a GluA1-based mechanism.

We found increased hippocampal GAD67 levels in VEH-treated *Nrg1* mutant mice compared to VEH WTs, which were decreased by chronic CBD treatment. In other studies, decreased PFC and hippocampal GAD67 protein and mRNA has been reported in individuals with schizophrenia (Akbarian and Huang, 2006). However, the *Nrg1 TM* mouse model has been proposed as a gain of function model (Long et al., 2015), thus elevated Nrg1 protein levels may increase GAD67 protein levels. Indeed, acute Nrg1 treatment has been found to increase GAD67 protein levels in a ketamine rat model of schizophrenia (Wang et al., 2014). Furthermore, recent work has shown that reduced hippocampal GAD67 binding of male poly I:C offspring can be normalized to control levels by chronic adolescent CBD (Osborne et al., 2019b) and CBD increased hippocampal GAD67 in both control and poly I:C female offspring (Osborne et al., 2019a).

In summary, this study suggests chronic adolescent CBD does not limit the development of schizophrenia-relevant behaviors in a *Nrg1* mouse model in young adulthood, and may therefore not be a potent preventative therapeutic candidate for patients harboring this mutation. Nonetheless, an increase in social behaviors after chronic adolescent CBD does suggest some therapeutic potential, perhaps for the treatment of social withdrawal. Future research should expand on our findings and consider also testing female mice and other CBD dosing regimes. Other genetic schizophrenia model systems should be considered also as CBD continues to show promise as an intervention for schizophrenia-relevant behaviors in other preclinical model systems (Osborne et al., 2017, 2019a; da Silva et al., 2020).

## Data availability statement

The original contributions presented in this study are included in the article/supplementary material, further inquiries can be directed to the corresponding authors.

## Ethics statement

This animal study was reviewed and approved by the Western Sydney University Animal Care and Ethics Committee (ARA: A13298 and A11746).

## Author contributions

GV, RC, KW-G, CSW, and TK designed the research. GV, SB, and RC performed the experiments. GV and RC wrote the manuscript. TK, CSW, and KW-G funded the research. All authors reviewed the manuscript prior to submission.

## Abbreviations

*NRG1*/*Nrg1*: *neuregulin 1*
CBD: cannabidiol
THC: Δ^9^ -tetrahydrocannabinol
OF: open field
SI: social interaction
PPI: prepulse inhibition
FC: fear conditioning
ITI: inter-trial interval
i.p: intraperitoneal
RM: repeated measures
SEM: standard error of means
WT: wild type-like.
